# Comparison of marker-based and center-of-pressure-based approaches for calculating the margin of stability

**DOI:** 10.3389/fspor.2025.1571994

**Published:** 2025-06-05

**Authors:** Cloé Dussault-Picard, Romain Tisserand, Claire Robidou, Yosra Cherni

**Affiliations:** ^1^ Ecole de Kinésiologie et des Sciences de L'activité Physique, Faculté de Médecine, Université de Montréal, Montréal, QC, Canada; ^2^ Laboratoire de Neurobiomécanique et Neuroréadaptation de la locomotion (NNL), Centre de recherche Azrieli du CHU Sainte Justine, Montréal, QC, Canada; ^3^ Université de Poitiers, ISAE-ENSMA, CNRS, PPrime, Poitiers, France; ^4^ Université de Poitiers, CNRS, CeRCA, Poitiers, France; ^5^ Institut de Génie Biomédical, Faculté de Medecine, Université de Montreal, Montréal, QC, Canada; ^6^ Centre Interdisciplinaire de Recherche sur le Cerveau et L'apprentissage (CIRCA), Faculté de Médecine, Université de Montréal, Montréal, QC, Canada

**Keywords:** balance, center of pressure, walking, stability, force platform

## Abstract

**Introduction:**

The margin of stability (MoS) is a widely used biomechanical measure of dynamic stability during gait, typically computed as the distance between the extrapolated center of mass (xCoM) and the center of pressure (CoP). According to Hof's model, the CoP-based approach is considered the preferred approach for defining where the xCoM is relative to the BoS and calculating the MoS. However, marker-based approaches often need to be used in research and clinical settings due to practical constraints and the lack of standardization in marker selection introduces variability in MoS estimates. This study aimed to assess the difference between different marker-based approaches and the CoP-based approach.

**Methods:**

Using an open-access dataset of 30 healthy adults walking at a self-selected speed, MoS was calculated continuously during the stance phase in both the anteroposterior (AP) and mediolateral (ML) directions. Various marker-based approaches were evaluated, including commonly used markers (AP: HEEL, TOE; ML: HEEL, ANKLE, M5, MID) and a novel approach using the most anterior (for AP MoS) or most lateral (for ML MoS) marker in contact with the ground at each time point (AP: MOST ANTERIOR; ML: MOST LATERAL). Differences were quantified using paired *t*-tests with statistical parametric mapping and root mean square differences (RMSD) relative to the CoP-based approach.

**Results:**

Results showed that the MOST ANTERIOR approach had the closest agreement with the CoP-based approach for AP MoS (RMSD = 47.04 mm), while the HEEL marker provided the closest agreement with the CoP-based approach for the ML MoS estimates (RMSD = 17.93 mm).

**Conclusion:**

These findings highlight the importance of marker selection in MoS analysis and suggest that specific marker configurations, particularly those grounded in foot-ground contact for the AP-MoS, provide closest estimates relative to the CoP-based approach. This study offers evidence-based recommendations for improving consistency and comparability in future MoS studies using marker-based approaches.

## 1 Introduction

Various methods have been employed to assess stability during human locomotion (1). These methods span from ordinal scale clinical assessments [e.g., the Berg Balance Scale (2) and the Functional Gait Assessment (3)] to biomechanical measures derived from a simple mechanical system [e.g., the margin of stability (MoS) (4)]. The MoS is one of the most widely used metrics to describe the instant mechanical stability of the body configuration during pathological (5) and non-pathological (6) gait. The MoS represents the minimum distance between the extrapolated center of mass (xCoM) and the boundaries of the base of support (BoS) (Equation 1).


(1) MoS=BoS–xCoM

The MoS was introduced by Hof and colleagues in 2005, and is based on the traditional linearized inverted pendulum model (4). The MoS is theoretically related to the minimal external impulse required to destabilize the body, as modeled by the inverted pendulum framework (7). The xCoM combines the CoM position (*COM*) and its velocity (*COM*) divided by the pendulum's eigen frequency, i.e., the square root of gravity (*g* *=* 9.81 m/s^2^) divided by the pendulum length *(l*) (Equation 2).

(2) $$xCoM=COM+\;\frac{COM}{\sqrt{\frac{g}{l}}}$$

**Figure 1:**
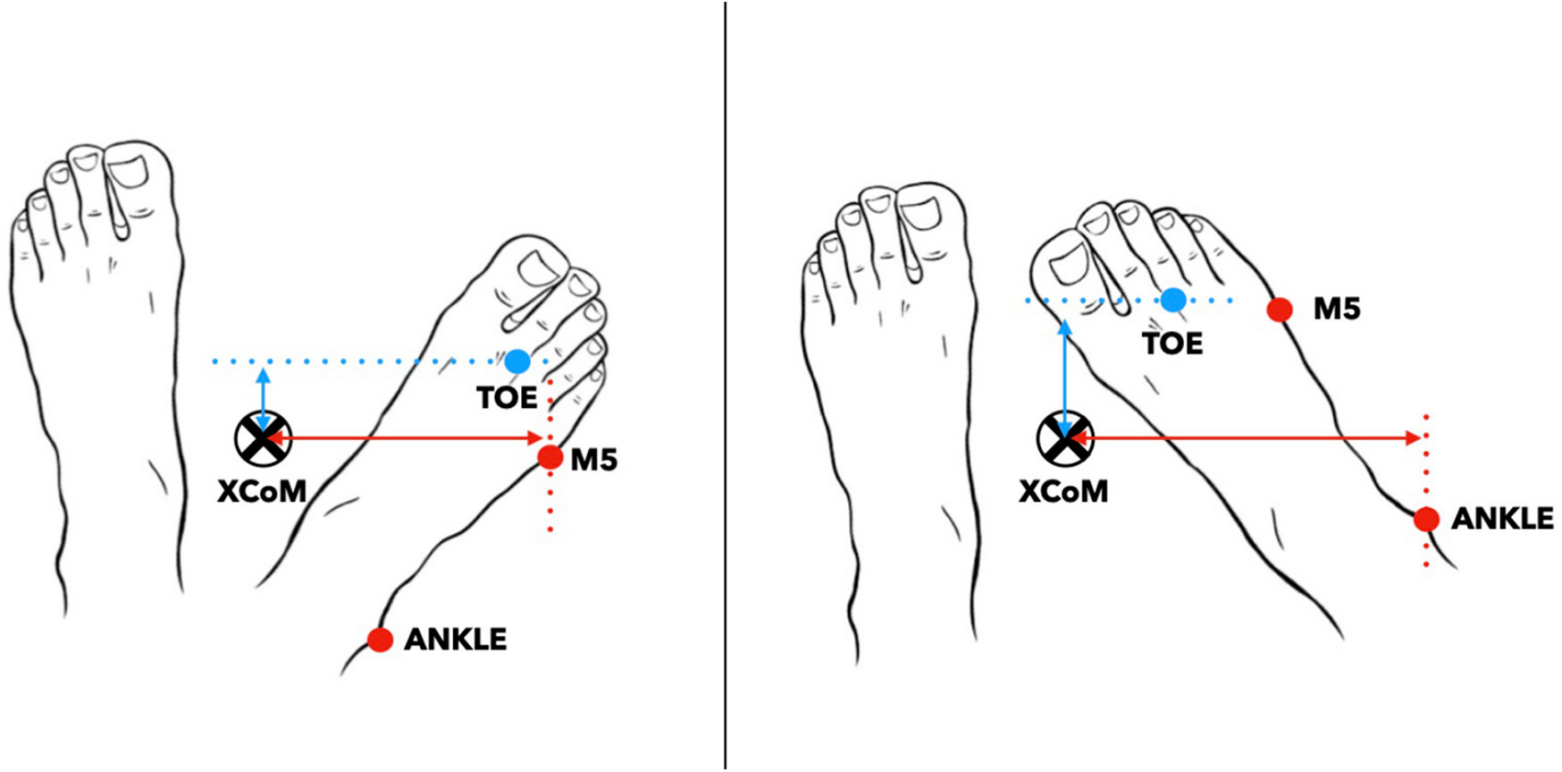
Representation of the most lateral marker during foot rotation between the fifth metatarsal (M5) and lateral malleolar (ANKLE) markers. The mediolateral margin of stability is the difference between the extrapolated center of mass (xCOM) and the most lateral marker. The anteroposterior margin of stability is the difference between xCOM and the most anterior marker, which is always the second metatarsal head marker (TOE). The mediolateral margin of stability is the difference between the extrapolated center of mass (xCOM) and the most lateral marker.

The MoS calculation relies not only on the accurate estimation of the xCoM but also on the precise definition of the BoS boundaries. It can be calculated in the anteroposterior (AP) and the mediolateral (ML) directions of the stance phase. The BoS boundaries' definition in previous studies are either based on the center of pressure (CoP) localization (8, 9) or foot markers (10–12). According to Hof's model, the CoP-based approach is considered the preferred method for defining the BoS and calculating the MoS, as the CoP represents the point of application of the ground reaction force acting on the CoM, which in turn influences the xCoM. However, some researchers have turned to marker-based approaches due to practical limitations, such as the unavailability of force plates or the challenges of using them in certain pathological populations, where multiple walking trials are often needed to capture enough valid steps on the platforms. Even in well-equipped laboratories, the limited number and surface area of force plates (typically one or two) may require numerous trials to record sufficient steps, making marker-based methods more efficient for collecting larger datasets in fewer passes. The boundaries of the BoS using marker-based approaches are heterogeneous across studies, which make interpretation and comparison of MoS results challenging (see a summary of the different approaches used in Supplementary Table S1). Although the definition of AP boundaries seems to be almost standardized, based on the toe marker (TOE) (anterior boundary) or heel marker (HEEL) (posterior boundary), the definition of the lateral boundary of the BoS remains heterogeneous (5). The lateral boundary of the BoS has been described with the fifth metatarsal marker (M5), the lateral malleolar marker (ANKLE), or the mid-point between M5 and ANKLE. Uncertainty persists regarding the markers defining the BoS boundaries at different instants of the stance phase (7). It has been suggested that using the midpoint on the virtual line relating M5 and ANKLE would have an advantage, compared to using ANKLE or M5, in considering the foot orientation rather than only a point (11, 13). This could be particularly relevant in individuals with consequent internal or external foot rotation (example in Figure 1). Another inconsistency is that, regardless of the approach chosen, the marker representing the BoS boundary is often placed on a foot boundary that is not always in contact with the ground (e.g., the ANKLE during late stance). This would limit the MoS accuracy because the CoP can only move underneath a body part in contact with the ground to restore stability in the event of balance perturbations. Thus, it can be hypothesized that the best representation of the CoP using marker-based approaches is achieved by using the most anterior (for the AP boundary) or the most lateral (for the ML boundary) marker that is fixed on a physical boundary of the foot that is in contact with the ground at the instant when the MoS is calculated. To date, no study has investigated which of the marker-based approaches provides the most accurate estimation of the MoS compared to the CoP-based approach.

This study aimed to assess differences in AP and ML MoS measures resulting from different marker-based approaches compared to the CoP-based approach. It was hypothesized that selecting the point located at the outermost boundary of the BoS [i.e., using the most anterior (for the AP) or the most lateral (for the ML)] marker that is in contact with the ground would provide a measure that is closer to the CoP-based approach and more representative of the effective MoS compared to previously established approaches.

## 2 Materials and methods

### 2.1 Participants

This study used an open-access dataset, including 30 healthy participants (16M/14F) aged between 21 and 41 years old (14). Participant characteristics are presented in Table 1.

**Table 1:** Participant characteristics.

Characteristics	Mean (SD)
*n*	30
Age	27.97 (5.59)
Height (m)	1.73 (0.92)
Body mass (kg)	68.17 (11.06)

### 2.2 Procedure

#### 2.2.1 Recordings

Each participant was instructed to walk at comfortable speed (mean speed (m/s) = 1.15 ± 0.10) along 10-m of a flat laboratory surface with walking shoes provided for the experiment (14). The laboratory, measuring 16 meters in length, ensured adequate space for participants to accelerate and decelerate outside the 10-meter measurement zone, minimizing the influence of these transitions on the recorded data (14). Several walking trials were performed. All participants were equipped with 63 reflective markers following the Conventional Gait Model (v.2.5) (15). Marker trajectories were collected with 18 optoelectronic cameras (Vicon System®, Oxford, UK, 100 Hz).

#### 2.2.2 Data processing

The c3d files were exported from the database (14) and further processed in MATLAB (vR2022b, Mathworks Inc., USA) using the open-source biomechZoo toolbox (v.1.9.10) (16) and custom codes. The force platform data was used to identify foot strike and foot off events. Then, walking trials were partitioned into individual stance phase. Considering the natural asymmetry in able-bodied gait (17), stance phases of both legs were included in the analysis. For each participant, 7 stance phases were used for MoS calculations. This number corresponds to the minimum number of valid stance phases on the force platform that was consistently available across all participants.

#### 2.2.3 Margin of stability calculations

The continuous MoS was calculated during the stance phase in the AP and ML directions (4). The CoM position estimated from the Conventional Gait model (v.2.5) was used (i.e., mass-weighted average of all segment CoMs). The anterior direction of walking was described as the vector of the walking direction whereas the lateral direction of walking was described as the vector perpendicular to the anterior direction of walking. The Supplementary Table S1 summarizes the different marker-based approaches used in the previous literature to calculate the MoS.

The AP MoS was calculated at each time point of the stance phase following 3 different marker-based approaches (HEEL, TOE, MOST ANTERIOR):

1) **HEEL:** Using **HEEL** as the posterior limit of the BoS.
2) **TOE:** Using **TOE** as the anterior limit of the BoS.
3) **MOST ANTERIOR:** Using the most **anterior** marker between **HEEL** and **TOE**. The foot part to which the most anterior marker is attached had to be in contact with the ground. For instance, **HEEL** was chosen if the forefoot (**TOE**) was elevated (at foot strike), whereas **TOE** was selected if the heel was elevated (during late stance).

The ML MoS was calculated at each time point of the stance phase following 5 marker-based approaches (ANKLE, M5, MID, HEEL, MOST LATERAL):

1) **ANKLE:** Using **ANKLE** as the lateral limit of the BoS.
2) **M5:** Using **M5** as the lateral limit of the BoS. By identifying the most lateral marker as the lateral limit of the BoS.
3) **MID:** Using the midpoint of the virtual line relating **ANKLE** and **M5** as the lateral limit of the BoS.
4) **HEEL:** Using **HEEL** as the lateral limit of the BoS.
5) **MOST LATERAL:** Using the most **lateral** marker between **ANKLE** and **M5**. The foot part to which the most lateral marker is attached had to be in contact with the ground. For instance, **ANKLE** was chosen if the midfoot/forefoot (**M5**) was elevated (at foot strike), whereas **M5** was selected if the heel was elevated (during late stance).

Both AP and ML MoS were also calculated at each time point of the stance phase following the CoP-based approach (COP). The AP and ML CoP position in the global reference frame was calculated as followed:

$$AP\;CoP_{Global}=\;\left(\frac{M_{x_{Local}}}{F_{z_{Local}}}\right)+\;T_{y}$$

$$ML\;CoP_{Global}=\;-\left(\frac{M_{y}}{F_{z_{Local}}}\right)+\;T_{x}$$

where *M*_y_ and *M_x_* are the ground reaction moments of the AP and ML axes, Fz, is the ground reaction forces, and *T_x_*, and *T_y_* are the translation to translate the CoP measure of the local reference frame of the force plate into the global reference frame.

All calculations were performed using MATLAB (vR2024b, Mathworks Inc., USA). The MoS curves were time-normalized to 100 data points, corresponding to percentages of the stance phase. MoS curves were averaged across participants, for each calculation approach.

### 2.3 Statistical analysis

To test our hypothesis, paired *t*-tests were conducted to assess differences between each marker-based AP MoS calculation approach (HEEL, TOE, and MOST ANTERIOR) and the CoP-based approach using the Statistical Parametric Mapping (SPM) toolbox (18) and custom-made Matlab scripts (spm1d.stats.ttest_paired function, spm1d v.M.0.4.11). The same tests were conducted to evaluate the differences between each of the three marker-based ML MoS calculation approaches (ANKLE, M5, MID, HEEL, and MOST LATERAL) with the CoP-based approach. The level of significance was adjusted for the paired *t*-test following a Bonferroni correction (*n* = 3 for AP MoS and *n* = 5 for ML MoS) to account for multiple comparisons (19). The SPM-based analysis captures differences over time, offering insight into how closely the marker-based curves follow the shape and magnitude of the CoP-based curve (representativeness). The significant clusters [i.e., multiple adjacent points of the SPM{t} curve exceeding the critical threshold computed based on Random Field Theory (18)] were identified, and their corresponding *p*-values were reported. The mean Cohen's d (*d*) effect size was calculated for each significant cluster (20). Only clusters lasting 5% or more were discussed (21). The effect size below 0.2 were considered very small, 0.2–0.5 as small, 0.5–0.8 as medium, 0.8–1.0 as large, and those above 1.0 as very large effects (20).

In addition, Pearson correlation analyses were performed to evaluate the within-participant similarity between the temporal profiles of each marker-based approach and the CoP-based approach. For each participant, the Pearson correlation coefficient was computed between the two corresponding time series, resulting in one correlation value per participant. These individual correlation coefficients (*r*) were then Fisher z-transformed to allow parametric statistical analysis, and a one-sample *t*-test was conducted to determine whether the mean Fisher z-value significantly differed from zero. The mean *r* and the resulting *p*-value were reported for each correlation test. The correlation was interpreted as negligible (*r* = 0.00–0.10), weak (*r* = 0.10–0.39), moderate (*r* = 0.40–0.69), strong (*r* = 0.70–0.89), and very strong (*r* = 0.90–1.00) (22).

To quantify the general agreement in magnitude between each marker-based approach with the CoP-based approach across the stance phase, the root mean square difference (RMSD) was calculated for each participant. The, mean RMS across all participants was computed to obtain a group-level estimate of the overall agreement between the approaches.

## 3 Results

### 3.1 Anteroposterior margin of stability

**Figure 2:**
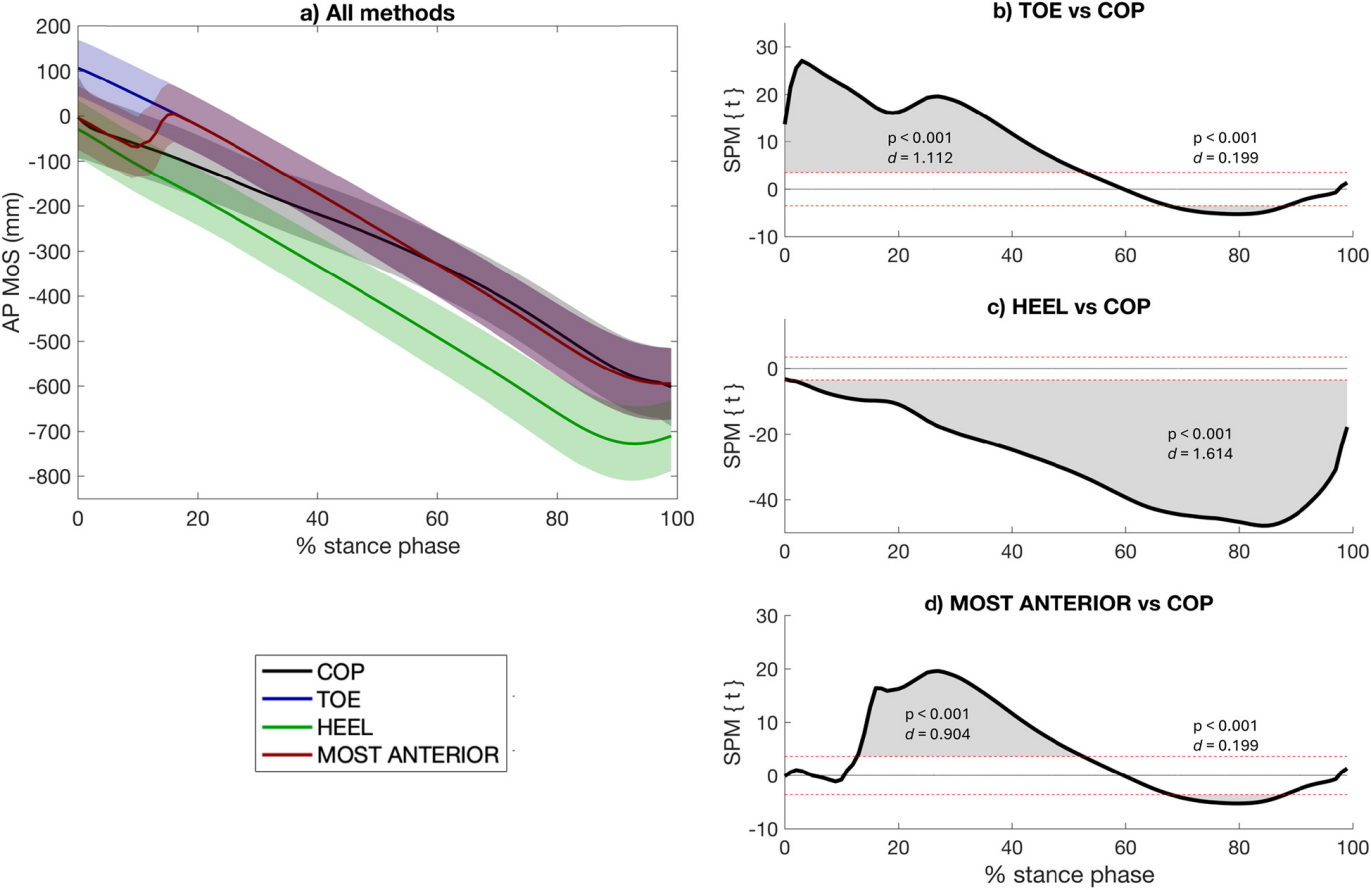
Anteroposterior (AP) margin of stability (MoS) calculated using the two most widely used marker-based approaches in the literature (i.e., TOE, HEEL), the approach proposed in this study (i.e., MOST ANTERIOR), and the center-of-pressure (COP)-based approach to describe the anterior limit of the base of support **(a)**. A negative AP MoS refers to an extrapolated center of mass that is in front of the anterior limit of the BoS. The differences between each marker-based approach with the CoP-based approach are presented [statistical parametric mapping (SPM) paired *t*-test, *p* < 0.05] **(b–d)**. The red dashed lines indicate the critical thresholds for statistical significance. Values above or under these lines indicate statistically significant differences between the compared approaches at that specific point in the stance phase.

The marker-based calculation approach HEEL yielded smaller MoS than the COP throughout the entire stance phase (0%–100%, *p* < 0.001, *d* = 1.614) (Figures 2a,c). The TOE approach led to higher MoS than the COP during the first part of the stance phase (0%–54%, *p* < 0.001, *d* = 1.112), and smaller MoS during the end of stance phase (68%–89%, *p* < 0.001, *d* = 1.614) (Figures 2a,b). When using the MOST ANTERIOR approach, the MoS is smaller than the COP at the end of the stance phase (69%–89%, *p* < 0.001, *d* = 0.199) whereas it is higher during the single leg stance (13%–53%, *p* < 0.001, *d* = 0.904) (Figures 2a,d). The MOST ANTERIOR approach had the best overall agreement with the COP: HEEL RMSD = 131.38 ± 17.90 mm; TOE RMSD = 62.25 ± 14.86 mm; MOST ANTERIOR RMSD = 47.04 ± 13.94 mm (Table 2). All marker-based approaches had very strong correlation with the CoP-based approach (*r* > 0.987, *p* < 0.001) (Table 2). The stance phase group mean RMSD of each AP approach and their correlation with the COP approach are presented in Supplementary Figure S1.

**Table 2:** Mean stance phase root means square difference and correlation results of the margin of stability for each marker-based approach compared to the center of pressure-based approach.

Anteroposterior margin of stability	Root means square difference	Correlation	
	Mean ± SD (mm)	*r*	*p*
HEEL	131.38 ± 17.90	0.990	<0.001
TOE	62.25 ± 14.86	0.994	<0.001
MOST ANTERIOR	47.04 ± 13.94	0.987	<0.001
Mediolateral margin of stability	Mean ± SD (mm)	*r*	*p*
M5	56.24 ± 6.75	0.749	<0.001
MID	45.40 ± 5.20	0.721	<0.001
ANKLE	34.88 ± 6.42	0.681	<0.001
HEEL	17.93 ± 8.32	0.739	<0.001
MOST LATERAL	53.80 ± 6.22	0.883	<0.001

### 3.2 Mediolateral margin of stability

**Figure 3:**
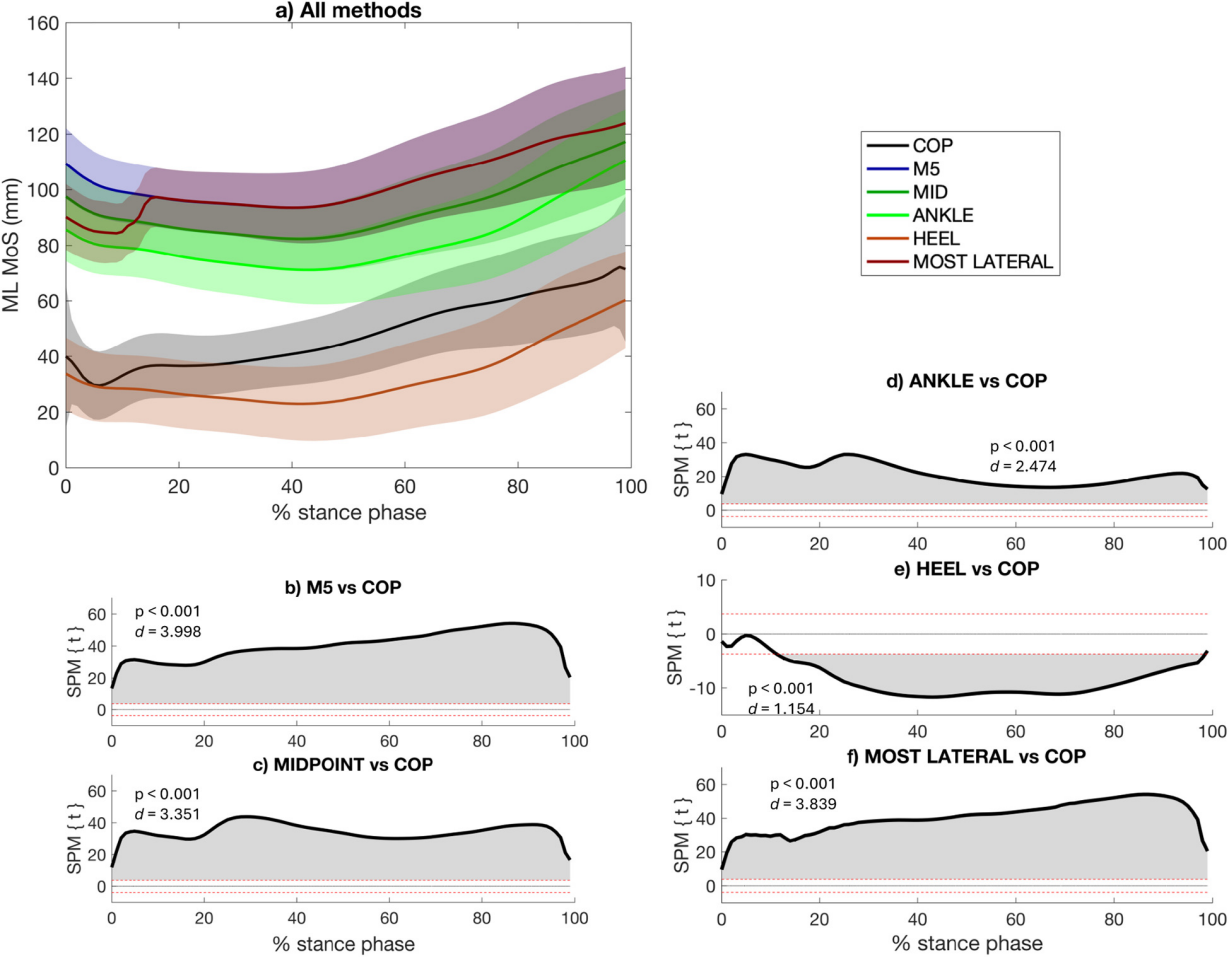
Mediolateral (ML) margin of stability (MoS) calculated using the three most widely used marker-based approaches in the literature (i.e., ANKLE, M5, MID, HEEL), the approach proposed in this study, (i.e., MOST LATERAL), and the center-of-pressure (COP)-based approach to describe the lateral limit of the base of support **(a)**. A negative ML MoS refers to an extrapolated center of mass that is more lateral than the BoS. The differences between each marker-based approach with the CoP-based approach are presented [statistical parametric mapping (SPM) paired *t*-test, *p* < 0.05] **(b–f)**. The red dashed lines indicate the critical thresholds for statistical significance. Values above or under these lines indicate statistically significant differences between the compared approaches at that specific point in the stance phase.

Among the the 5 marker-based calculation approaches, 4 yielded higher MoS than the COP throughout the entire stance phase: COP vs. M5 (0%–100%, *p* < 0.001, *d* = 3.998) (Figures 3a,b), COP vs. MID (0%–100%, *p* < 0.001, *d* = 3.351) (Figures 3a,c), COP vs. ANKLE (0%–100%, *p* < 0.001, *d* = 2.474) (Figures 3a,d), COP vs. MOST LATERAL (0%–100%, *p* < 0.001, *d* = 3.839) (Figures 3a,f), whereas the HEEL approach yielded lower MoS than the COP throughout almost the entire stance phase (12%–99%, *p* < 0.001, *d* = 1.154) (Figure 3e). The HEEL approach had the best overall agreement with the COP: M5 RMSD = 56.24 ± 6.75 mm; MID RMSD = 45.40 ± 5.20 mm; ANKLE RMSD = 34.88 ± 6.42 mm, HEEL RMSD = 17.93 ± 8.32 mm, MOST LATERAL RMSD = 53.80 ± 6.22 mm (Table 2). The MOST LATERAL approach had the strongest correlation with the CoP-based approach (*r* = 0.883, *p* < 0.001) (Table 2). The stance phase group mean RMSD of each ML approach and their correlation with the COP approach are presented in Supplementary Figure S1.

## 4 Discussion

### 4.1 Summary

The aim of this study was to assess differences between various marker-based approaches for calculating AP and ML MoS compared to the CoP-based approach. Based on their closer agreement with the CoP-based approach throughout the stance phase, the marker-based MOST ANTERIOR and HEEL approaches proved to be the closest to the CoP approach for calculating AP and ML MoS continuously during the stance phase, respectively.

### 4.2 The effect of the calculation approach

The BoS is a key component of the MoS calculation, defined as the area between the feet during walking outlined by the points of contact with the ground, an area that determines the possibilities of moving the CoP. As expected, using marker-based approaches, the results showed that the marker chosen to describe the BoS boundaries affects the resulting AP and ML MoS values (Figures 2, 3, respectively). These results are in line with the perspective article by Curtze et al. (7), which highlighted that the definition of the BoS boundaries can influence the resulting MoS (7). However, the authors did not quantify the extent of this influence on the measurements, nor did they compare different marker-based approaches (7), gaps that the present study have addressed. The findings also complement those of Havens et al. (23), who have reported that biases in the MoS value can be introduced by the approach used to estimate the CoM dynamics (23). In the current study, the CoM dynamics were computed using the gold-standard approach (23), which involves a mass-weighted average of all body segment CoMs. Together, this study and that of Havens et al. (23) demonstrate that MoS values are highly sensitive to methodological choices: the present study highlights the influence of BoS definition (marker-based vs. CoP-based), while Havens et al. (23) emphasized the impact of CoM dynamics estimation (23). This underscores the importance of avoiding comparisons between studies that used different calculation approaches and the adoption of a standardized approach.

For the AP MoS, the results showed that the proposed approach, referred to as “MOST ANTERIOR”, had the overall closest agreement with the CoP-based approach. This is likely because the MOST ANTERIOR marker-based approach selects the most anterior marker in contact with the ground at each instant of the stance phase, typically the heel at the beginning of stance and the toe toward the end, which effectively mirrors the path followed by the CoP throughout the stance phase (7). However, it is worth noting that studies investigating the MoS at specific instants of the stance phase, as is common in previous literature (e.g., at initial contact), should carefully consider the most appropriate marker to use depending on the timing of the analysis. For instance, the results have shown that the MOST ANTERIOR approach is more in agreement with the CoP approach at initial contact, whereas it might not be the most appropriate during the single leg stance (13%–53%) (Figures 2a,d).

Concerning the ML MoS, it was also hypothesized that the proposed methods, referred to as “MOST LATERAL”, may be more appropriate to account for the effective BoS throughout the stance phase. In this study, 5 marker-based approaches were compared to the CoP-based approach. In one hand, the MOST LATERAL approach did not prove to be the marker-based method that most closely reflects the CoP-based approach for calculating ML MoS across the stance phase. In fact, the results show that using the MOST LATERAL approach tends to provide an overly lateral estimation of the BoS compared to the CoP that typically moves from the lateral to the medial part the foot during stance phase (24), leading to an overestimation of the ML MoS (Figures 3a,f). On the other hand, in terms of temporal profile, the MOST LATERAL approach showed the highest correlation with the CoP-based approach (Table 2), likely because the ML weight shift captured by the CoP-based approach during the first 20% of the stance phase coincides with the moment the MOST LATERAL algorithm switches from the ANKLE to the M5 marker (i.e., to respect the condition of using a marker fixed on a foot part that is in contact with the ground) (Figure 3a**)**. Among the 5 marker-based methods, the HEEL approach appears to minimize the lateral overestimation of the BoS, by using a marker that is positioned closer to the center of the foot and more in line with the typical position of the CoP. However, using HEEL approach relies on a single marker, which does not capture foot orientation and may limit the accuracy of the BoS in some situations, especially in population with foot deformities. This limitation was less critical in the present study, as all participants were healthy.

It is worth noting that in Hof's foundational paper (4), the MoS was calculated at initial foot contact (e.g., heel strike), where the CoP is intentionally placed a certain distance medial or lateral to the xCoM to allow for adjustments in gait, such as turning or stopping (5). This specific timing was also chosen because it corresponds to the moment when the distance between the CoP and xCoM is minimal, potentially representing the point of greatest instability. While the MoS provides an instantaneous assessment of stability at any given moment, applying it beyond initial contact is still valid, but it requires acknowledging that the lateral boundary of the BoS may evolve over time. This is particularly relevant when using marker-based definitions of the BoS, which may not fully capture these temporal changes. Supporting this, van Leeuwen et al. (25) showed that the CoP is actively modulated via ankle moments to compensate for mediolateral foot placement errors (25), suggesting that it reflects dynamic balance control (i.e., weight shifting) rather than anatomical foot limits. Thus, while the CoP-based approach is often considered the preferred method based on Hof's model (4), it may not fully capture the anatomical boundaries of the BoS in the ML direction. Thus, marker-based methods may provide a more stable and representative estimate of the lateral BoS, especially in clinical populations with atypical foot positioning or deformities.

Using marker-based approaches, the literature reveals an important heterogeneity in ML MoS calculation, which makes comparisons between studies and populations difficult (5). For example, in children with cerebral palsy, some studies opted for ANKLE to describe the lateral boundary of the BoS (26–28), while others used the MID (29). In populations with in- or out-toeing gait such as cerebral palsy (30, 31), both approaches may misestimate the BoS (32). Similarly, using M5 to describe the lateral BoS boundary may not be relevant in individuals with a medial foot rotation. Thus, future research should investigate which approach between the HEEL and the MOST LATERAL is more suitable for pathological populations with foot deformities or atypical foot orientations, as it may offer a more stable and anatomically neutral estimate of the lateral BoS boundary across a variety of gait patterns.

In the present study, significant differences were found between marker-based and CoP-based MoS estimates at certain points of the stance phase, although the temporal profiles showed moderate to very strong mean correlations. The mean RMSD across the stance phase between marker-based and CoP-based approaches ranged from 47.04 to 131.38 mm in the AP direction and from 17.93 to 46.24 mm in the ML direction. Using a CoP-based approach, De Jong et al. (33) reported a mean test-retest difference of 3.4 ± 14.1 mm for AP MoS and 26.7 ± 49.7 mm for ML MoS at heel strike in healthy adults (33). Therefore, the differences observed in this study between marker-based approaches and the CoP-based approach may represent clinically meaningful changes in stability, as they exceed the natural variability typically observed between sessions. This variability may be influenced by both gait fluctuations and the measurement noise inherent to motion capture and force plate systems. These findings highlight the need for caution when comparing MoS values across studies that use different calculation approach, as methodological differences can lead to variations that exceed typical test-retest variability but may not reflect actual differences in dynamic stability.

### 4.3 Limitations

This study has some limitations. First, while this study focused on the absolute agreement between marker-based and CoP-based MoS approaches, it did not assess whether different approaches yield consistent relative outcomes (e.g., between-group or between-condition effects). Future studies should investigate whether marker-based approaches maintain validity in detecting such differences across populations or experimental conditions. Second, expanding the study to include pathological populations and a wider age range would help generalize findings and enhance their clinical relevance. Third, the participant age range is too narrow, limiting the applicability of the results to other age groups. Finally, the data were collected from participants who walked while wearing their shoes, meaning that the markers were placed on top of the shoe. This setup could potentially lead to an overestimation of the lateral position of the markers located at midpoint between ankle and the fifth metatarsal (MIDPOINT approach), and fifth metatarsal (M5 approach).

### 4.4 Conclusion

This study provides reference measurements regarding the differences introduced by marker-based approaches compared to the CoP approach for calculating the MoS. The reported RMSDs could be used as reference values for researchers aiming to compare their results with studies that have employed different marker configurations. In addition, this study provides recommendations to improve the agreement of future marker-based studies with CoP-based studies and to help standardize current calculation practices. When a marker-based approach must be used across the entire stance phase, the findings suggest that, for values closer to those obtained with the CoP-based approach, the most anterior marker from the part of the foot in contact with the ground should be used for calculating the AP MoS. For calculating the ML MoS, the heel marker should be chosen, at least in population presenting no foot deformities or altered foot orientation.

## Data Availability

Publicly available datasets were analyzed in this study. This data can be found here: https://springernature.figshare.com/articles/dataset/3D_motion_analysis_dataset_of_healthy_young_adult_volunteers_walking_and_running_on_overground_and_treadmill/25592865?backTo=%2Fcollections%2F3D_motion_analysis_dataset_of_healthy_young_adult_volunteers_walking_and_running_on_overground_and_treadmill%2F7056797&file=45621447.
