# Supplementary material for: Comparison of marker-based and center-of-pressure-based approaches for calculating the margin of stability
Source: Front Sports Act Living. 2025 Jun 5;7:1571994. doi: 10.3389/fspor.2025.1571994 (PMC12179766; doi:10.3389/fspor.2025.1571994)
Supplement: Supplementary file 2 [file Image2.pdf]

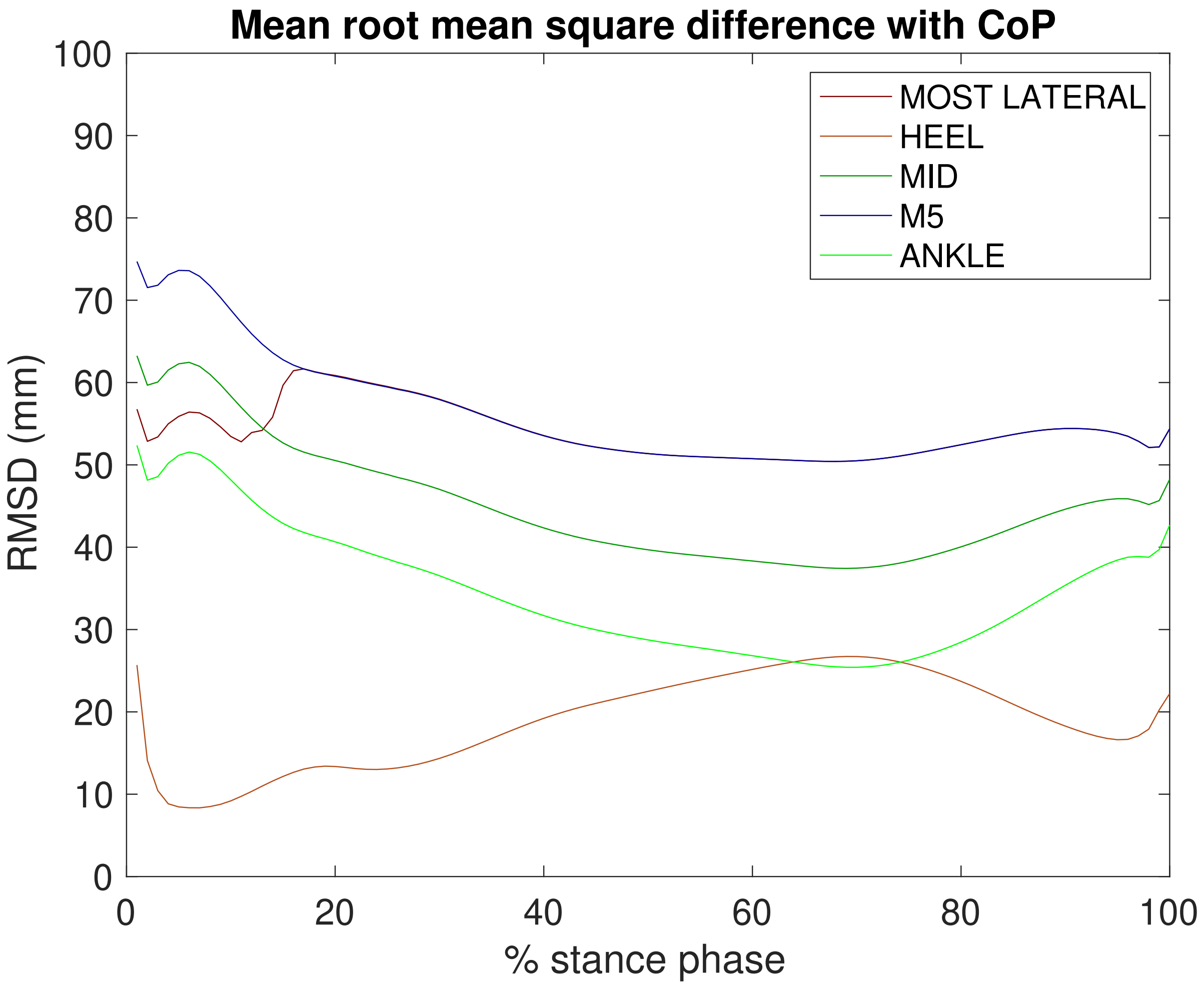

Supplementary figure 2. The continuous group mean root mean square difference (RMSD) with the center of pressure (CoP)-based approach of each anteroposterior approach (MOST LATERAL, HEEL, MID, M5, ANKLE).
