# Supplementary material for: Comparison of marker-based and center-of-pressure-based approaches for calculating the margin of stability
Source: Front Sports Act Living. 2025 Jun 5;7:1571994. doi: 10.3389/fspor.2025.1571994 (PMC12179766; doi:10.3389/fspor.2025.1571994)
Supplement: Supplementary file 3 [file Table1.docx]

**Supplementary table 1.** Literature review on the different approaches used to calculate the margin of stability

| **Study information** | | | **BoS definition** | | **Population** | |
| --- | --- | --- | --- | --- | --- | --- |
| **Year** | **Authors** | **Tittle** | **AP marker** | **ML marker** | **Groups (n)** | **Age (years old)** |
| 2011 | Curtze, C., Hof, A. L., Postema, K., & Otten, B. | Over rough and smooth: Amputee gait on an irregular surface | TOE | Midpoint: Calcaneus-M2 | A: 18 | A: 55.6 ± 9.5 |
| 2011 | Carty, C. P., Mills, P., & Barrett, R. | Recovery from forward loss of balance in young and older adults using the stepping strategy | TOE |  | E: 31 HYA: 16 | E: [65.0 - 80.0] HYA: [20.0 - 35.0] |
| 2012 | Young, P. M. M., & Dingwell, J. B. | Voluntary changes in step width and step length during human walking affect dynamic margins of stability | TOE | Lateral heel marker | HYA: 13 | HYA: [18.0 - 35.0] |
| 2012 | Young, P. M. M., Wilken, J. M., & Dingwell, J. B. | Dynamic margins of stability during human walking in destabilizing environments | TOE | Lateral heel marker | HYA: 12 | n/s |
| 2012 | Süptitz, F., Karamanidis, K., Catalá, M. M., & Brüggemann, G. | Symmetry and reproducibility of the components of dynamic stability in young adults at different walking velocities on the treadmill | HALLUX |  | HYA: 11 | HYA: 25.5 ± 2.1 |
| 2013 | Hak, L., Van Dieën, J. H., Van Der Wurff, P., Prins, M. R., Mert, A., Beek, P. J., & Houdijk, H. | Walking in an unstable environment: Strategies used by transtibial amputees to prevent falling during Gait | ANKLE | ANKLE | HA: 9 TA: 10 | HA: 37.0 ± 11.4 TA: 38.8 ± 14.6 |
| 2013 | Hak, L., Houdijk, H., Van Der Wurff, P., Prins, M. R., Mert, A., Beek, P. J., & Van Dieën, J. H. | Stepping strategies used by post-stroke individuals to maintain margins of stability during walking | ANKLE | ANKLE | HA: 9 PS: 10 | HA: 57.3 ± 7.2 PS: 60.8 ± 8.4 |
| 2013 | Hak, L., Houdijk, H., Beek, P. J., & Van Dieën, J. H. | Steps to take to enhance gait stability: The stride frequency, stride length, and walking speed on local dynamic stability and margins of stability | HEEL | ANKLE | HYA: 9 | HYA: 21.9 ± 1.8 |
| 2013 | Gates, D. H., Scott, S. J., Wilken, J. M., & Dingwell, J. B. | Frontal plane dynamic margins of stability in individuals with and without transtibial amputation walking on a loose rock surface |  | M5 | HYA: 15 TA: 13 | HYA: 22.0 ± 5.0 TA: 28.0 ± 4.0 |
| 2014 | Beltran, E. J., Dingwell, J. B., & Wilken, J. M. | Margins of stability in young adults with traumatic transtibial amputation walking in destabilizing environments |  | M5 | HYA: 13 TA: 9 | HYA: 24.8 ± 6.9 TA: 30.7 ± 6.8 |
| 2014 | Kao, P., Dingwell, J. B., Higginson, J. S., & Binder-Macleod, S. | Dynamic instability during post-stroke hemiparetic walking | TOE | M5 | HA: 9 PS: 9 | HA: 61.7 ± 10.0 PS: 60.8 ± 9.0 |
| 2014 | McCrum, C., Eysel-Gosepath, K., Epro, G., Meijer, K., Savelberg, H. H. C. M., Brüggemann, G., & Karamanidis, K. | Deficient recovery response and adaptive feedback potential in dynamic gait stability in unilateral peripheral vestibular disorder patients | TOE | n/s | HA: 17 UPVD: 17 | HA: 51.0 ± 8.0 UPVD: 49.0 ± 9.0 |
| 2015 | Rijken, N., Van Engelen, B., Geurts, A., & Weerdesteyn, V. | Dynamic stability during level walking and obstacle crossing in persons with facioscapulohumeral muscular dystrophy | HEEL | ANKLE | FSHD: 10 | FSHD: [43.0 - 68.0] |
| 2015 | Hak, L., Houdijk, H., Wurff, P., Prins, M., Beek, P., & Van DieëN, J. | Stride frequency and length adjustment in post-stroke individuals: Influence on the margins of stability | HEEL | ANKLE | PS: 10 | PS: [26 - 74] |
| 2015 | Hoogkamer, W., Bruijn, S. M., Sunaert, S., Swinnen, S. P., Van Calenbergh, F., & Duysens, J. | Toward new sensitive measures to evaluate gait stability in focal cerebellar lesion patients | Posterior boundary of the feet | Lateral boundary of the feet | HYA: 14 CL: 18 | HYA: 24.4 ± 3.5 CL: 24.4 ± 7.3 |
| 2016 | Peebles, A. T., Reinholdt, A., Bruetsch, A. P., Lynch, S. G., & Huisinga, J. M. | Dynamic margin of stability during gait is altered in persons with multiple sclerosis | TOE | M5 | HA: 20 MS1: 20 MS2: 20 | HA: 47.5 ± 7.8 MS1: 45.8 ± 8.6 MS2: 45.9 ± 8.7 |
| 2016 | Delabastita, T., Desloovere, K., & Meyns, P. | Restricted Arm Swing Affects Gait Stability and Increased Walking Speed Alters Trunk Movements in Children with Cerebral Palsy |  | ANKLE | TD: 24 CP: 26 | TD: [5.0 - 12.0] CP: [4.0 - 12.0] |
| 2016 | van Meulen, F. B., Weenk, D., van Asseldonk, E. H., Schepers, H. M., Veltink, P. H., & Buurke, J. H. | Analysis of balance during functional walking in stroke survivors | Midpoint: front of each foot | Lateral shoe position | PS: 10 | PS: 63.2 ± 8.9 |
| 2017 | Simon, A., Lugade, V., Bernhardt, K., Larson, N. A., & Kaufman, K. | Assessment of stability during gait in patients with spinal deformity—A preliminary analysis using the dynamic stability margin | M5 | Midpoint: M5- ANKLE | HYA: 12 SD: 17 | HYA: [23.2 - 27.1] SD: [23.8 - 50.4] |
| 2017 | Ghomian, B., Mehdizadeh, S., Aghili, R., Naemi, R., Jafari, H., Machado, J., Silva, L. F., Lobarinhas, P., & Saeedi, H. | Rocker outsole shoes and margin of stability during walking: A preliminary study | TOE | M5 | DB: 1 | DB: 50.0 |
| 2017 | Acasio, J., Wu, M., Fey, N. P., & Gordon, K. E. | Stability-maneuverability trade-offs during lateral steps |  | M5 | HYA: 10 | HYA: 25.6 ± 3.4 |
| 2017 | Martelli, D., Luo, L., Kang, J., Kang, U. J., Fahn, S., & Agrawal, S. K. | Adaptation of Stability during Perturbed Walking in Parkinson’s Disease | HALLUX | M5 | HA: 9 PD: 9 | HA: 64.7 ± 7.3 PD: 64.3 ± 7.4 |
| 2017 | Peebles, A. T., Bruetsch, A. P., Lynch, S. G., & Huisinga, J. M. | Dynamic balance in persons with multiple  sclerosis who have a falls history is altered compared to non-fallers and to healthy controls | TOE | TOE | HA: 27 MS: 55 | HA: 44.9 ± 9.9 MS: 45.9 ± 9.4 |
| 2018 | Guaitolini, M., Aprigliano, F., Mannini, A., Sabatini, A. M., & Monaco, V. | Effects of gait speed on the margin of stability in healthy young adults | M1 | M5 | HYA: 8 | HYA: [22.0 - 32.0] |
| 2018 | Havens, K. L., Mukherjee, T., & Finley, J. M. | Analysis of biases in dynamic margins of stability introduced by the use of simplified center of mass estimates during walking and turning | TOE | Lateral heel marker | HYA: 12 | HYA: 26.0 ± 3.0 |
| 2018 | Tisserand, R., Armand, S., Allali, G., Schnider, A., & Baillieul, S. | Cognitive-motor dual-task interference modulates mediolateral dynamic stability during gait in post-stroke individuals. |  | Midpoint: HEEL-M2 | HA: 10 PS: 12 | HA: 68.5 ± 4 PS: 58.0 ± 12.8 |
| 2018 | Sivakumaran, S., Schinkel-Ivy, A., Masani, K., & Mansfield, A. | Relationship between margin of stability and deviations in spatiotemporal gait features in healthy young adults | TOE | M5 | HYA: 11 | HYA: 24.0 ± 4.4 |
| 2018 | McCrum, C., Willems, P., Karamanidis, K., & Meijer, K. | Stability-normalised walking speed: a new approach for human  gait perturbation research | HALLUX |  | HYA: 18 | HYA: 24.4 ± 2.5 |
| 2019 | AminiAghdam, S., Griessbach, E., Vielemeyer, J., & Müller, R. | Dynamic postural control during (in)visible curb descent at fast versus comfortable walking velocity | HALLUX |  | HYA: 12 | HYA: 25.5 ± 4.7 |
| 2019 | Tracy, J. B., Petersen, D. A., Pigman, J., Conner, B. C., Wright, H. G., Modlesky, C. M., Miller, F., Johnson, C. L., & Crenshaw, J. R. | Dynamic stability during walking in children with and without cerebral palsy | TOE | n/s | CP: 15 TD: 14 | CP: 8.7 ± 2.4 TD: 9.1 ± 2.5 |
| 2019 | Lencioni, T., Carpinella, I., Rabuffetti, M., Cattaneo, D., & Ferrarin, M. | Measures of dynamic balance during level walking in healthy adult subjects: Relationship with age, anthropometry and spatio-temporal gait parameters | M5 | M5 | HA: 34 | HA: [21.0 - 71.0] |
| 2019 | Van Vugt, Y., Stinear, J., Davies, T. C., & Zhang, Y. | Postural stability during gait for adults with hereditary spastic paraparesis | Metatarsal marker of the stance foot | 2cm lateral to M2 | HA: 10 HSP: 10 | HA: 56.4 ± 16.0 HSP: 53.5 ± 11.5 |
| 2019 | Ohtsu, H., Yoshida, S., Minamisawa, T., Takahashi, T., Yomogida, S., & Kanzaki, H. | Investigation of balance strategy over gait cycle based on margin of stability | M5 | Medial HEEL | HYA: 30 | HYA: 21.2 ± 0.8 |
| 2019 | Arora, T., Musselman, K. E., Lanovaz, J. L., Linassi, G., Arnold, C., Milosavljevic, S., & Oates, A. | Walking Stability During Normal Walking and Its Association with Slip Intensity Among Individuals with Incomplete Spinal Cord Injury | n/s |  | HA: 16 ISCI: 20 | HA: 58.9 ± 17.1 ISCI: 60.0 ± 17.8 |
| 2019 | Major, M. J., McConn, S. M., Zavaleta, J. L., Stine, R., & Gard, S. A. | Effects of upper limb loss and prosthesis use on proactive mechanisms of locomotor stability |  | M5 | ULL: 10 | ULL: 50.0 ± 19.0 |
| 2020 | Herssens, N., Van Criekinge, T., Saeys, W., Truijen, S., Vereeck, L., Van Rompaey, V., & Hallemans, A. | An investigation of the spatio-temporal parameters of gait and margins of stability throughout adulthood | HEEL | M5 | HA: 105 | HA: [20.0 - 89.0] |
| 2021 | Ma, Y., Mithraratne, K., Wilson, N. C., Zhang, Y., & Wang, X. | Kinect V2-Based Gait Analysis for Children with Cerebral Palsy: Validity and Reliability of Spatial Margin of Stability and Spatiotemporal Variables | TOE | ANKLE | CP: 10 | CP: 6.4 ± 2.2 |
| 2021 | Rethwilm, R., Böhm, H., Haase, M., Perchthaler, D., Dussa, C. U., & Federolf, P. | Dynamic stability in cerebral palsy during walking and running: Predictors and regulation strategies |  | ANKLE | CP: 117 TD: 25 | CP: 11.0 ± 3.2 TD: 10.4 ± 2.5 |
| 2022 | Yamaguchi, T., & Masani, K. | Effects of age on dynamic balance measures and their correlation during walking across the adult lifespan | HEEL | M5 | HA: 151 | HA: [20.0 - 77.0] |
| 2024 | Sangeux, M., Viehweger, E., Romkes, J., & Bracht-Schweizer, K. | On the clinical interpretation of overground gait stability indices in children with cerebral palsy | TOE | M5 | TD: 20 CP: 20 | TD: [7.7 - 16.7] CP: [8.3 - 17.8] |
| 2024 | Wang, Z., Xie, H., & Chien, J. H. | The margin of stability is affected differently when walking under quasi-random treadmill perturbations with or without full visual support | HEEL | M5 | HYA: 20 | HYA: 22.6 ± 2.8 |

Summary of studies (n = 41) that have investigated either the antero-posterior (AP) or the mediolateral (ML) margin of stability (MoS). For each MoS calculation, the markers used to define AP and/or ML base of support were retrieved. The population (n, age, and pathology) included is describe. The age is reported by the range [min - max], or by the mean ± SD, according to how it was reported in the study. Empty boxes indicate a that the element was not assessed by the study. Population abbreviations are as follows: Amputees, AM; Cerebellar lesions, CL; Cerebral palsy, CP; Diabetes, DB; Elderly, E; Facioscapulohumeral muscular dystrophy, FSHD; Healthy adults, HA; Hereditary spastic paraparesis, HSP; Healthy young adults, HYA; Incomplete spinal cord injury, ISCI; Multiple sclerosis, MS; Multiple sclerosis without gait impairments, MS1; Multiple sclerosis with gait impairments, MS2; Parkinson disease, PD; Post-stroke, PS; Spinal deformity, SD; TA, Transtibial Amputees; Typically developing, TD; Upper limb loss, ULL; Unilateral peripheral vestibular disorder, UPVD. Other abbreviations: ANKLE, Lateral malleoli; HALLUX, Hallux; HEEL, Calcaneum; M5, 5^th^ metatarsal; TOE, 2^nd^ metatarsal.
